# Impact of Human Papillomavirus on microRNA-21 Expression in Oral and Oropharyngeal Cancer—A Systematic Review

**DOI:** 10.3390/ijms25158038

**Published:** 2024-07-23

**Authors:** Mario Kordic, Dinko Martinovic, Ema Puizina, Josko Bozic, Zeljko Zubcic, Emil Dediol

**Affiliations:** 1 Department of ENT and Maxillofacial Surgery, University Hospital Mostar, 88000 Mostar, Bosnia and Herzegovina; korda333@gmail.com; 2 Department of Maxillofacial Surgery, University Hospital of Split, 21000 Split, Croatia; dmartinovic@kbsplit.hr (D.M.); epuizina@kbsplit.hr (E.P.); 3 Department of Pathophysiology, University of Split School of Medicine, 21000 Split, Croatia; josko.bozic@mefst.hr; 4 Department of ENT, University Hospital of Osijek, 31000 Osijek, Croatia; zeljko.zubcic@mefos.hr; 5 Department of Maxillofacial Surgery, University Hospital Dubrava, 10000 Zagreb, Croatia

**Keywords:** oral cancer, oropharyngeal cancer, HPV, microRNA-21

## Abstract

Recently, microRNAs (miR) were identified to have potential links with oral squamous cell carcinoma (OSCC) and oropharyngeal squamous cell carcinoma (OPSCC) oncogenesis, specifically miR-21. Since HPV is a major risk factor for the development of these diseases, we aimed to search the literature regarding miR-21 expression in both HPV-positive and HPV-negative OSCC/OPSCC. The search was performed in the PubMed (MEDLINE), Scopus, Web of Science, and Cochrane electronic databases. The research question was as follows: Is there a difference in the tissue expression of miR-21 between patients with HPV-positive and those with HPV-negative OSCC/OPSCC? After conducting a meticulous search strategy, four studies were included, and they had a pooled sample size of 621 subjects with OSCC and/or OPSCC. Three studies did not find any significant difference in miR-21 expression between HPV-positive and HPV-negative OSCC/OPSCC. The findings of this systematic review showed that there are no differences in miR-21 expression between HPV-positive and HPV-negative OSCC/OPSCC. Nevertheless, it is worth noting that there are still insufficient studies regarding this important subject, because understanding how HPV influences miR-21 expression and its downstream effects can provide insights into the molecular mechanisms underlying OSCC/OPSCC development and progression.

## 1. Introduction

Oral squamous cell carcinoma (OSCC) ranks sixth in global malignancy prevalence and stands as the predominant malignancy within head and neck neoplasms [1]. It can be challenging to differentiate the incidence and prevalence of OSCC independently of oropharyngeal squamous cell carcinoma (OPSCC) as there is huge variation in the categorization of these cancers. OPSCC anatomically comprises cancers of the tonsils, base of the tongue, soft palate, and uvula. However, different authors and classifications categorize these anatomical locations regarding the OPSCC and OSCC differently. Nevertheless, due to their anatomical location, they can both significantly impact patients’ quality of life and necessitate comprehensive management. Moreover, substantial morbidity and mortality rates associated with OSCC/OPSCC present a significant burden on the global health system [2,3]. Although numerous etiological factors contribute to both OSCC and OPSCC pathogenesis, alcohol and tobacco consumption persist as paramount risk determinants [4]. Additional risk factors have been concurrently associated with other pathological conditions, including the existence of oral precancerous lesions such as leukoplakia, erythroplakia, and lichen planus, as well as infectious agents like HPV, HCV, and EBV [5]. Furthermore, 25 to 35% of all head and neck squamous cell carcinomas (HNSCC) have been shown to be associated with human papillomavirus (HPV), and these are mostly confined to the tonsils and the base of tongue, which should anatomically belong to the OPSCC [6,7]. Moreover, recent studies indicate that HNSCC associated with HPV represents a distinct subgroup, characterized by unique epidemiology, histopathological features, responses to chemotherapy and radiation, and clinical outcomes [8].

The OSCC and OPSCC oncogenesis process is highly complex and multifaceted as it encompasses genetic alterations, epigenetic modifications, and disturbances in the tumor microenvironment. While diverse therapeutic strategies, including surgery, chemotherapy, radiation, immunotherapy, and nanomedicine have been suggested for the prevention and treatment of OSCC/OPSCC, unraveling the mechanisms underlying these malignancies will aid in pinpointing therapeutic targets and prognostic indicators [3]. The principal genetic modifications implicated in carcinogenesis encompass alterations in tumor suppressors such as APC, p53, BRCA2, PTCH, NF1, VHL, Rb, BCL2, SWI/SNF, p16, CD95, ST5, YPEL3, ST7, and ST14, alongside oncogenes including Ras, jun, fas, erbA, abl, raf, gsp, sis, erbB, and fms [9,10]. Furthermore, their initiation and progression entail changes in DNA methylation, histone modifications, and non-coding RNA alterations, including microRNAs (miRNA) [11,12,13]. MicroRNAs (miRNAs) constitute a group of small endogenous non-coding RNAs that modulate various biological processes by regulating gene expression. The dysregulation of miRNAs enables tumor cells to acquire tumorigenic traits, including heightened proliferative signaling, the evasion of suppressive mechanisms, resistance to apoptosis, enhanced invasive and metastatic capabilities, and the stimulation of angiogenesis [14]. Recent studies have shown that the dysregulation of non-coding RNA is a negative prognostic survival factor [15]. Specifically, the expression of miR-21 appears to be altered in many neoplasms, and its differential expression in tumor tissues can represent a prognostic biomarker of survival [16].

The human miR-21 gene spans 3433 nucleotides and is situated within an intergenic region on chromosome 17q23.2 [17]. This gene features two transcription sites: T1 (the minor transcription site) and T2 (the primary transcription site). In the nucleus, RNA polymerase II catalyzes the transcription of primary miR-21 (~3.5 kb), which is subsequently transported to the cytoplasm. There, polymerase III releases the mature 22-nucleotide miR-21, which becomes integrated into and degraded by the miRNA–RISC complex. Alterations in mechanisms governing miR-21’s expression, transcription, transport, binding with RISC, and degradation processes may lead to the dysregulated bioregulation of miR-21 in tumor cells [18,19].

The most recent studies are pointing out that miR-21 could have both prognostic and diagnostic value for OSCC/OPSCC, as it was shown that its overexpression in the tumor tissue possibly plays an important role in its oncogenesis [16,20,21,22]. Furthermore, miR-21 has been studied in relation to HPV-positive cervical cancer, and there is evidence suggesting an association between them, as it was shown that miR-21 is upregulated in HPV-positive cervical cancer [23]. HPV infection can lead to the dysregulation of cellular processes, including gene expression, and this can contribute to the overexpression of miR-21. Moreover, studies have suggested that miR-21 may play a role in HPV-mediated carcinogenesis by affecting key cellular processes [24,25]. For instance, miR-21 can suppress the expression of tumor-suppressor genes like PTEN, PDCD4, and TPM1, which are involved in controlling cell growth and apoptosis [26]. Hence, HPV-positive OSCC/OPSCC differs from HPV-negative OSCC/OPSCC in several molecular and clinical aspects, indicating that the HPV-positive cancer may represent a separate and distinct tumor entity [27,28]. Furthermore, HPV16, the most prevalent HPV type in cervical squamous cell carcinomas, is also the most common type found in HPV-positive OSCC/OPSCC [29]. Additionally, since the reticulated tonsillar lymphoepithelium is believed to be particularly susceptible to HPV infection and the associated tumorigenesis, immunohistochemical staining for p16 serves as a surrogate marker for HPV infection in OPSCC, with HPV-positive tumors exhibiting p16 overexpression [30,31].

Henceforth, since recent publications showed that miR-21 could have prognostic value for OSCC/OPSCC, and HPV is a major risk factor for developing these diseases, by carrying out a systematic review of the data in the literature, we aim to provide the most up-to-date data on the differential expression of oral cancer miR-21 in correlation with HPV positivity.

## 2. Methods

### 2.1. Study Design

This systematic review was performed according to the Preferred Reporting Items for Systematic Reviews and Meta-Analyses (PRISMA) guidelines. Furthermore, the protocol of the search strategy, the inclusion criteria, and the search outcomes were registered in PROSPERO under the number CRD42024521596 prior to the screening.

Primarily, a systematic review with a meta-analysis was planned. However, after meticulous research of the available literature, due to the small number of studies which were eligible and the high heterogeneity of both the immunohistochemical and statistical tests conducted, a meta-analysis was not possible.

### 2.2. Inclusion and Exclusion Criteria

The inclusion criteria were as follows: studies that indicated and reported data comparisons of the tissue expression of miR-21 between HPV-positive and HPV-negative OSCC/OPSCC.

The exclusion criteria were as follows: studies published in a non-English language; studies which did not report miR-21 expression in both HPV-positive and HPV-negative OSCC/OPSCC; studies with a high risk of bias.

All prospective and retrospective studies, as well as RCTs, that evaluated differences in the tissue expression of miR-21 in oral carcinoma in correlation with HPV positivity were considered. Henceforth, the PICO question was as follows: “Is there a difference in the tissue expression of miR-21 between patients with HPV-positive and those with HPV-negative oral cancer and/or oropharyngeal cancer?”

### 2.3. Search Strategy and the Selection Process

All considered studies were detected through meticulous bibliographic searches of electronic databases conducted by two researchers (D.M. and M.K.). The filter relating to the language of the publications was used, and all studies in a language other than English were excluded. The search was performed in the PubMed (MEDLINE), Scopus, Web of Science, and Cochrane electronic databases, and the last literature search was conducted on 15 April 2024. Moreover, a search of the gray literature and on Google Scholar was also performed, and the bibliographic sources of previous systematic reviews on the subject were correspondingly investigated.

The following terms were used to search the databases: “miR-21”; “oral cancer”; “microRNA”; “OSCC”; “HPV”; “human papilloma virus”; “oropharyngeal cancer”. The terms were combined using the commands “AND” or “OR”.

All duplicates were eliminated manually. The identified articles were independently evaluated and scrutinized by 2 reviewers (D.M. and M.K.). Articles were selected in two phases. In the first phase, both reviewers independently screened the titles and abstracts for relevance. Any disagreements were resolved by E.D. In the second phase, both reviewers independently performed full-text reviews (D.M. and M.K.). Disagreements at this stage were also resolved by E.D.

### 2.4. Data Extraction

The following data were extracted to a spreadsheet in Microsoft Excel, version 6.2.14 (Microsoft Office, Redmond, DC, USA): author, year of publication, country, diagnosis, study sample size, test used to determine miR-21, statistical method for the association between HPV and miR-21, results.

## 3. Results and Discussion

**Figure 1:**
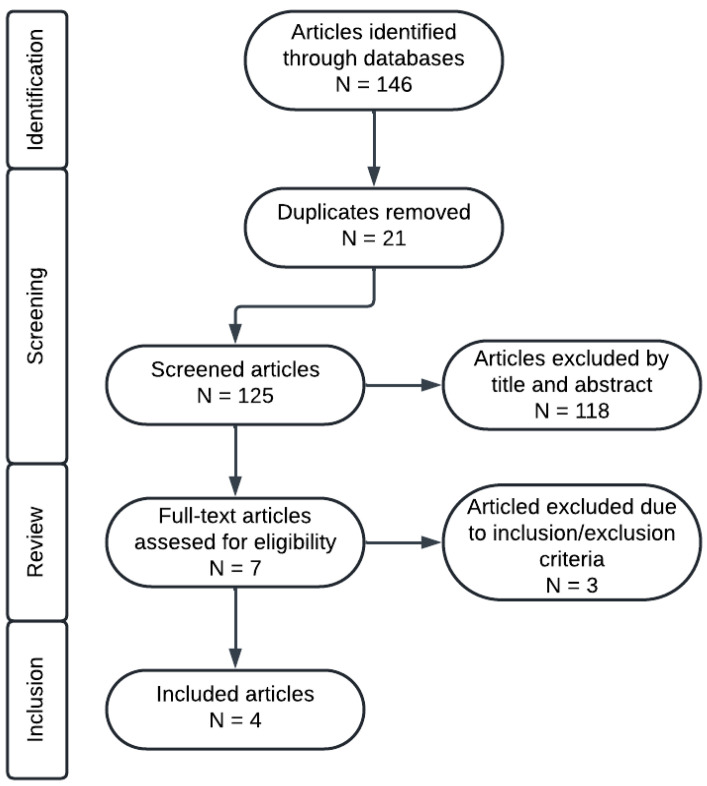
Flowchart of the selected studies.

Using the aforementioned search strategy, there were initially 146 records retrieved (Figure 1). During the selection process, 21 duplicates were removed and 125 articles underwent title and abstract reviews. This resulted in seven articles for full-text evaluation. Of these, three articles were excluded due to not fulfilling the inclusion criteria. Hence, four articles were included in this study.

The included studies were relatively recently published and they had a pooled sample size of 621 subjects with OSCC and/or OPSCC (Table 1). 

Three studies used commercial kits for quantitative real-time reverse transcription PCR (qRT-PCR) for determining miRNA-21 expression in the tumors (Table 2) [32,33,34]. All of these studies used different kits for the RNA isolation, reverse transcription, and PCR analyses (Table 2). One study used a commercial kit for in situ hybridization (ISH) using a specific miR-21 probe [35].

The pooled HPV-positive sample size was 135 and the HPV-negative sample size was 486 (Table 3). Only the results of Orosz E. et al. showed a higher expression of miRNA-21 in HPV-positive oral cancer compared to the HPV-negative OPSCC [32]. However, they used an ANOVA test to compare miRNA-21 expression between several biopsies which were taken at different distances from the tumor. On the other hand, Mehterov N. et al. used a Mann–Whitney U test to compare miRNA-21 expression on the largest sample size and they did not find a significant difference [33]. Likewise, Simic I. et al. used the same method, but on a smaller sample, and found the same result of no significant difference in miRNA-21 expression between HPV-positive and HPV-negative OSCC/OPSCC [34]. Lastly, Ho Ko Y. et al. dichotomized their results of miRNA-21 expression into “higher” and “lower” expression categories [35]. Using chi-square analysis, they did not find a significant difference.

Recent studies are pointing out that miR-21 expression could possibly have prognostic value regarding the survival of patients with OSCC/OPSCC, and the latest meta-analysis showed that high expression of miR-21 was characterized by the worsening of overall survival [36]. Moreover, another recent meta-analysis showed that even the circulating levels of miR-21 could be used as a diagnostic biomarker for OSCC/OPSCC [16]. All this evidence suggests that there is a complex and close relationship between OSCC/OPSCC pathophysiology and miR-21. On the other hand, it is well established that HPV manipulates host cellular pathways, including those involved in microRNA regulation, and consequently promotes oncogenesis.

**Figure 2:**
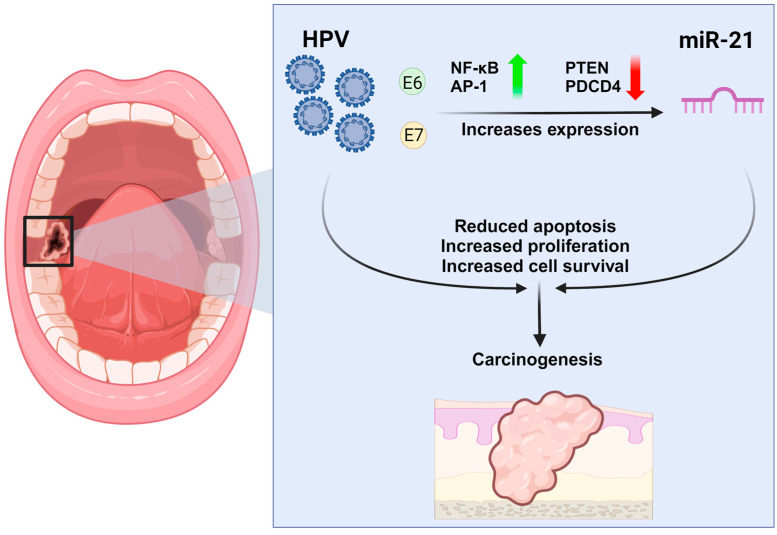
Biological correlation between HPV and miR-21 with its impact on OSCC/OPSCC development.

HPV is a known risk factor for developing malignant disease, specifically OSCC, and especially regarding OPSCC [37,38]. The major oncogenic proteins of HPV, E6 and E7, play critical roles in modulating cellular processes [39]. E6 targets and degrades the tumor suppressor protein p53, while E7 interacts with and inactivates the Rb tumor suppressor protein [40]. These actions disrupt normal cell cycle regulation and promote cellular proliferation. Moreover, HPV can activate transcription factors that directly promote the expression of miR-21. Likewise, E6 can enhance the activity of transcription factors like AP-1 and NF-κB, which are known to stimulate miR-21 transcription [41,42,43,44]. Additionally, E6 and E7 can activate cellular signaling pathways that promote miR-21 expression. For instance, activation of the PI3K/Akt pathway by HPV oncoproteins can enhance miR-21 expression, contributing to increased cell proliferation and survival [45,46]. This is achieved through the Akt downstream activation of transcription factors, such as the previously mentioned AP-1 and NF-κB, and consequently increases miR-21 transcription and subsequent expression and tumorigenesis. Furthermore, by inhibiting tumor suppressors like PTEN (phosphatase and tensin homolog) and PDCD4 (programmed cell death 4), HPV indirectly promotes the upregulation of miR-21 [47]. These tumor suppressors are normally targeted and inhibited by miR-21, creating a positive feedback loop that sustains oncogenic signaling [48,49]. PTEN can indirectly affect miR-21 levels through its role in the PI3K/Akt pathway. Both PTEN and PDCD4 are direct targets of miR-21, which leads to their degradation and translational inhibition. This subsequently leads to increased cell proliferation and reduced apoptosis and facilitates cancer progression (Figure 2). HPV can also alter the expression and activity of proteins involved in miRNA biogenesis, such as Dicer and Drosha [50,51]. Drosha cleaves the primary miR to precursor miR which are later processed with the Dicer to a mature miR. Changes in these protein enzymes can lead to the increased processing of miR-21 and other oncogenic miRNAs.

However, the results of our systematic review regarding the difference in miR-21 expression between HPV-positive and HPV-negative OSCC/OPSCC partially showed that there is no significant difference between them. While miR-21 expression in OSCC/OPSCC was found to be higher in all of the included studies, Mehterov et al. did not find any statistically significant difference in miR-21 expression between HPV-positive and HPV-negative OSCC [33]. Furthermore, the same results were confirmed in the study by Simic et al. with a similar methodology but on a smaller sample size and mixed OSCC and OPSCC [34]. While Ho Ko et al. used qualitative dichotomized miR-21 expression in OSCC/OPSCC, they still found the same result that there are no differences between HPV-positive and HPV-negative cancer [35]. In contrast, the only study that did find a significant difference, Orosz et al., did not compare miR-21 expression from just the OPSCC tissue, but also from the surrounding mucosa [32]. They took several biopsies, from the tumor itself as well as from several locations on the surrounding mucosa with different distances from the tumor. Additionally, they did not conduct a post hoc test after the ANOVA analysis to show where exactly the statistically significant differences were found. Hence, their result could be deemed of “smaller” significance for this systematic review.

It is imperative to contextualize miR-21 within the broader biological framework, as it is extensively acknowledged as an oncogenic microRNA, frequently exhibiting elevated expression across a wide spectrum of malignancies, including cervical, breast, lung, hepatic, and colorectal cancers [14,19,23]. MiR-21 is instrumental in facilitating oncogenic processes such as cellular proliferation, invasion, metastasis, and the evasion of apoptosis [43,44,45,47]. Although our review did not find significant variations in miR-21 expression between HPV-positive and HPV-negative cases, the involvement of miR-21 in critical oncogenic pathways highlights its potential as a therapeutic target and a prognostic biomarker. Elucidating the specific mechanisms through which miR-21 contributes to HPV-associated cancers could pave the way for more effective diagnostic and therapeutic strategies.

It is important to highlight that the aforementioned results of miR-21 expression in oral cancer are in line with the outcomes of several studies which evaluated miR-21 expression in cervical cancer [52,53,54]. It was shown by several studies that miR-21 expression is higher in cervical cancer and that there is no significant difference in expression between HPV-positive and HPV-negative cervical cancer [23,52]. Furthermore, the results of the in vitro study by Han et al. suggested that the epithelial–mesenchymal transition, the initial process of epithelial malignant tumor invasion, could be linked with miR-21 upregulation [55]. They found that HPV possibly induces miR-21 to promote epithelial stromal transformation and the tumor progression of cervical cancer cells. Furthermore, high levels of miR-21 expression in HPV-positive cervical cancer have been associated with more aggressive disease and poorer prognosis [56]. Nevertheless, it seems that even though HPV is also a major risk factor for developing cervical cancer, similar to the results of OSCC/OPSCC studies, there are no significant differences in miR-21 tissue expression. This suggests that even though the oncogenesis process is different between the HPV-positive and HPV-negative cancer, it is possible that miR-21 does not play a major role in that difference. Hence, while it could be deemed a possibly good prognostic and diagnostic tool for oral cancer, it is not possible to differentiate cancers which are induced by HPV from those which are not.

Furthermore, it is significant to remark that it has been established that HPV is more associated with OPSCC than OSCC [57]. According to the literature, in some regions, HPV-positive OPSCC accounts for up to 70% of all OPSCC [58]. The proportion of HPV-positive OPSCC is especially on the rise in the USA and Europe due to the drop in alcohol and tobacco usage, which are the primary etiological risk factors for HPV-negative OPSCC. On the other hand, HPV-positive OSCC is not as highly represented in the total number of OSCCs [38]. Several important factors influence this disproportion between HPV-positive OSCC and OPSCC. Firstly, HPV is sexually transmitted and consequently aligns more closely to the oropharynx than the oral cavity. HPV is much more transient in the oral cavity due to the epithelial lining, which is less conductive for HPV [59]. The stratified epithelium of the oral cavity lacks the same level of crypts and lymphoid tissue compared to those present in the oropharynx. Subsequently, HPV infection is much more persistent in the oropharynx due to its reticulated epithelium and crypts, which trap the virus. Hence, this systematic review includes both OPSCC and OSCC due to the poor differentiation between these two entities according to different studies and classifications. Two of the included studies in this systematic review had both patients with OPSCC and OSCC, while one study had only patients with OSCC, and the last study had only OPSCC patients. However, regarding miR-21’s association with HPV, it is worth noting that there was no significant correlation in the study which included only OPSCC patients.

Lastly, it is important to note that all of the included studies did not use the same methodology to determine miR-21 expression. Except one study, which used the ISH, all other included studies used qRT-PCR. It is well established that that qRT-PCR has high sensitivity and high specificity for determining miR expression, while ISH has both low specificity and low sensitivity [60,61]. The ISH conducted in the aforementioned study was enhanced using locked nucleic acid (LNA), which is advantageous regarding specificity and sensitivity; however, it is still a semi-quantitative method, and the signal may not directly correlate with the miR-21 expression level [62,63]. LNA-ISH is much more suitable for studying miR-21 expression patterns and localization within tumor tissues. In contrast, qRT-PCR is ideal for the precise quantification of miR-21 expression. Three different methods of qRT-PCR were used in the included studies. SYBR Green qRT-PCR relies on the use of a fluorescent dye which binds to double-stranded DNA during the amplification process and is consequently susceptible to nonspecific primer–dimer formation, which can lead to false-positive signals. On the contrary, Taqman qRT-PCR uses sequence-specific probes, which gives it high specificity and reduces the risk of detecting nonspecific amplification products. Similarly, LNA qRT-PCR uses LNA-modified probes for enhanced binding affinity and stability, which also gives it high specificity and sensitivity. Henceforth, the heterogeneity in the methodologies of the included studies could possibly interfere with the comparison of their outcomes regarding miR-21 tissue expression in both HPV-positive and HPV-negative OSCC/OPSCC.

Besides HPV status, various other factors may also impact miR-21 expression, such as smoking, alcohol intake, and genetic predispositions. Smoking and alcohol consumption are well-established risk factors for OSCC/OPSCC and have been shown to modify microRNA expression profiles [64,65,66]. Moreover, genetic predispositions can influence miRNA expression and function, potentially confounding the observed correlations between miR-21 expression and HPV status [67,68]. Unfortunately, the studies included in our review did not consistently report data on these potential confounding factors, which consequently limits our ability to fully adjust for their effects. Future studies should aim to comprehensively account for these variables to provide a more accurate evaluation of miR-21 expression patterns.

Another limitation of our review is the dependence on a limited number of studies, encompassing a total of 621 subjects. This restricted sample size may influence the robustness and generalizability of our findings, particularly the conclusion that there are no differences in miR-21 expression between HPV-positive and HPV-negative OSCC/OPSCC. Small sample sizes can lead to diminished statistical power, thereby increasing the risk of Type II errors. Consequently, our findings should be interpreted with care, and further studies with larger sample sizes are necessary to validate our results and ensure their applicability to broader populations.

## 4. Conclusions

In conclusion, the findings of this systematic review showed that there are no differences in miR-21 expression between HPV-positive and HPV-negative OSCC/OPSCC. Nevertheless, it is worth noting that there are still insufficient studies regarding this important subject, because understanding how HPV influences miR-21 expression and its downstream effects can provide insights into the molecular mechanisms underlying OSCC/OPSCC development and progression. Targeting miR-21 or its downstream pathways could potentially be explored as a therapeutic strategy in HPV-positive OSCC/OPSCC cancer. While our review provides preliminary insights into miR-21 expression in HPV-positive and HPV-negative OSCC/OPSCC, the limited number of studies and sample size underscores the necessity for larger, more comprehensive studies to confirm these findings and explore their clinical implications.

## Figures and Tables

**Table 1:** Included studies’ general characteristics.

Author	Year of Publication	Country	Diagnosis	Study Sample Size
Orosz E. et al. [32]	2020	Hungary	OPSCC	25
Mehterov N. et al. [33]	2022	Bulgaria	OSCC	353
Simic I. et al. [34]	2023	Croatia	OSCC, OPSCC	76
Ho Ko Y. et al. [35]	2014	Republic of Korea	OSCC, OPSCC	167

**Abbreviations:** OSCC—oral squamous cell carcinoma; OPSCC—oropharyngeal squamous cell carcinoma.

**Table 2:** Tests used to determine miRNA expression.

Reference	miRNA Expression Method	Commercial Kits Used
[32]	LNA qRT-PCR	Aurum Total RNA mini kit (BioRad, Hercules, CA, USA) miRCury LNA Universal RT microRNA PCR Kit (Qiagen, Hilden, Germany) miRCury LNA miRNA PCR assays (Qiagen, Hilden, Germany)
[33]	SYBR Green qRT-PCR	PAXgene Tissue miRNA Kit (Qiagen, Hilden, Germany) miScript Reverse Transcription Kit (Qiagen, Hilden, Germany) SYBR^®^ Green PCR Kit (Qiagen, Hilden, Germany)
[34]	Taqman probe qRT-PCR	TaqMan Advanced miRNA Assays (Applied Biosystems, Waltham, MA, USA) TaqMan Advanced miRNA cDNA Synthesis Kit (Applied Biosystems, Waltham, MA, USA) CFX96 Touch Real-Time PCR Detection System (BioRad, Hercules, CA, USA)
[35]	LNA-ISH	miRCury LNA miRNA Detection FFPE (Exiqon, Vedbaek, Denmark) microRNA ISH Optimization Kit2 (Exiqon, Vedbaek, Denmark)

**Abbreviations:** qRT-PCR—quantitative real-time reverse transcription polymerase chain reaction; ISH—in situ hybridization; LNA—locked nuclei acid.

**Table 3:** HPV-positive and -negative samples and the results of the included studies.

Reference	HPV-Positive Sample Size	HPV-Negative Sample Size	Mean miR-21 Expression	Statistical Method Used	miR-21 Expression in Regard to HPV
[32]	8	17	Higher	ANOVA	miRNA-21 higher expression in HPV-positive tumors
[33]	73	280	Higher	Mann–Whitney U test	No difference
[34]	18	58	Higher	Mann–Whitney U test	No difference
[35]	36	131	Higher	Chi-square	No difference

**Abbreviations:** HPV—human papillomavirus; ANOVA—analysis of variance.
